# Sustained Palmitoylethanolamide Infusion Restores Incentive Motivation and Synaptic Plasticity in the Tg2576 Mouse Model of Alzheimer’s Disease

**DOI:** 10.3390/cells15080669

**Published:** 2026-04-09

**Authors:** Anna Panuccio, Zuleyha Nihan Yurtsever, Debora Cutuli, Giacomo Giacovazzo, Davide Decandia, Daniel Tortolani, Eugenia Landolfo, Sergio Oddi, Mauro Maccarrone, Laura Petrosini, Roberto Coccurello

**Affiliations:** 1 European Center for Brain Research, IRCCS Santa Lucia Foundation, 00143 Rome, Italy; 2 Department of Psychology, University Sapienza of Rome, 00185 Rome, Italy; 3 Department of Veterinary Medicine, University of Teramo, 64100 Teramo, Italy; 4 Department of Biotechnological and Applied Clinical Sciences, University of L’Aquila, 67100 L’Aquila, Italy; 5 Institute for Complex Systems (ISC), National Council of Research (CNR), 00185 Rome, Italy

**Keywords:** palmitoylethanolamide, Alzheimer’s disease, motivation-driven behavior, peroxisome proliferator-activated receptor alpha, conditioned place preference, neuroplasticity, prefrontal cortex, entorhinal cortex, brain-derived neurotrophic factor, Tg2576 mouse model

## Abstract

**Highlights:**

**What are the main findings?**

- Chronic infusion of palmitoylethanolamide (PEA) restores motivation-driven behavior in Tg2576 mice, assessed by the conditioned place preference paradigm.
- PEA upregulates PPAR-α and BDNF expression in the prefrontal cortex of Tg2576 mice, and rescues dendritic complexity in the entorhinal cortex and dentate gyrus.

**What are the implications of the main findings?**

- The activation of PEA-PPARα-BDNF signaling pathway may contribute to reverse non-cognitive symptoms of Alzheimer’s disease, such as motivational and anhedonia-like symptoms.
- Lipid-based neuromodulation via PEA offers a potentially novel therapeutic route to restore synaptic and behavioral plasticity in rewarding-motivational circuits affected in early AD.

**Abstract:**

Alzheimer’s disease (AD) is increasingly recognized as a disorder not only of cognition but also of motivation and emotional regulation. Apathy and anhedonia often precede memory deficits, implicating early dysfunction in reward-related circuits. This study investigated whether chronic infusion of palmitoylethanolamide (PEA), a lipid-derived PPARα agonist, could restore motivational behavior and dendritic plasticity in the Tg2576 mouse model of AD. The motivational behavior of mice that received sustained-release PEA pellets for 6 months was assessed by using the conditioned place preference (CPP) paradigm. Morphological and molecular analyses were conducted in the entorhinal cortex (EC), dentate gyrus (DG), and prefrontal cortex (PFC). In Tg2576 mice, PEA significantly rescued CPP performance, increased basal dendritic spines in WT mice in the EC, and both basal and apical dendritic expression in EC and DG from Tg2576 mice, and upregulated the expression of both PPAR-α and brain-derived neurotrophic factor (BDNF) in the PFC. Interestingly, the BDNF increase occurred even in the absence of baseline deficits, suggesting a trophic-enhancement effect. These findings suggest that the PEA-PPARα-BDNF axis may be a potential mechanism for restoring motivation and synaptic integrity in an AD-like mouse model. Lipid-based neuromodulation may therefore offer novel therapeutic routes for addressing non-cognitive symptoms and affective circuitopathy in neurodegenerative diseases.

## 1. Introduction

Alzheimer’s disease (AD) is traditionally defined as a progressive neurodegenerative disorder characterized by memory decline, cognitive impairment, and neuropathological hallmarks, such as amyloid-β (Aβ) deposition and tau hyperphosphorylation. However, beyond cognitive deterioration, patients also frequently exhibit early neuropsychiatric symptoms, including apathy, anhedonia, anxiety, agitation, aggression, and emotional blunting [1,2,3]. These non-cognitive dimensions of AD may also be ascribed to the potential pathogenetic role of microbiota [3], and contribute substantially to disease burden, accelerate cognitive decline, and worsen prognosis. Emotional and motivational dysfunctions may precede overt memory deficits, suggesting that the neural systems governing reward, affect, and motivation are disrupted early in the disease process [4,5]. Among the earliest affected cortical regions in AD, the entorhinal cortex (EC) has a pivotal position at the intersection of memory, emotion, and motivation [6,7,8]. The EC serves as a bidirectional relay between the hippocampus, amygdala, and prefrontal cortex (PFC), regions essential for associative learning, emotional evaluation, and goal-directed behavior. Early tau pathology and synaptic loss within the EC have been strongly correlated with both mnemonic decline and affective dysregulation [7,9]. Functional neuroimaging in humans and electrophysiological studies in rodents demonstrate that EC activity integrates affective salience and motivational significance of stimuli [9]. In mouse models of AD, EC dysfunction contributes to the reduced exploratory drive, apathy, social and emotional withdrawal, paralleling the motivational flattening observed in patients [2,10,11]. Among transgenic models, Tg2576 mice express a human amyloid precursor protein variant that drives a progressive, age-dependent increase in amyloid burden accompanied by synaptic dysfunction and behavioral alterations [12]. This model is particularly suitable for investigating motivational and affective disturbances that emerge alongside, and sometimes precede, overt cognitive impairment [13].

These findings support the emerging view that AD involves a broad emotion-motivation circuitopathy, in which EC–hippocampal–prefrontal interactions deteriorate alongside classical memory networks. Emotional and motivational behaviors depend on the integrity of synaptic connectivity within limbic networks. In transgenic AD models, chronic Aβ accumulation and hyperphosphorylated tau protein disrupt dendritic spine morphology and density in both the hippocampus and EC [14,15,16]. These structural alterations parallel deficits in reward-based tasks, including conditioned place preference (CPP), which require intact motivational and affective processing [17]. As a Pavlovian behavioral paradigm, particularly a contextual associative learning task, CPP is widely used to assess the motivational valence of reward-associated contextual stimuli. Using the CPP paradigm, it is possible to probe memory, affective processing, and the neural circuit dynamics involved in associative learning [17,18]. CPP engages a distributed neural network involving the hippocampus, PFC, and EC, integrating contextual encoding, decision-making during retrieval [19], and multimodal memory processing [20,21] From this view, the EC can be considered a primary interface between the hippocampus and neocortical areas. Deficits in CPP performance have been observed in neurodegenerative conditions such as AD [17], as well as in various neuropathological contexts, including neurodevelopmental disorders such as autism spectrum disorder [22,23] and following chronic stress exposure or systemic inflammation [24,25]. Notably, in all of these conditions, the neural circuits underlying associative learning, reward processing, and contextual memory can be disrupted.

At the molecular level, these impairments can be associated with downregulation of brain-derived neurotrophic factor (BDNF), a key mediator of neuronal survival, synaptic plasticity, and emotional regulation [26,27]. BDNF mimetics or TrkB agonists are involved in CPP expression in experimental models of reward or memory impairment by enhancing synaptic efficacy and plasticity [28]. Reduced BDNF signaling in AD correlates with the loss of dendritic spines and synaptic markers, particularly in the EC, dentate gyrus (DG), and PFC [29]. Moreover, BDNF deficiency contributes not only to cognitive decline but also to anhedonia and depressive-like behavior, indicating its dual role in cognitive and emotional resilience [27,30].

Palmitoylethanolamide (PEA) is an endogenous lipid belonging to the N-acylethanolamide (NAE) family, known for its anti-inflammatory, neuroprotective, immunomodulatory, and neurotrophic properties [31,32,33,34]. PEA acts primarily by activating peroxisome proliferator-activated receptor alpha (PPARα), a nuclear receptor that regulates lipid metabolism, inflammatory gene expression, and mitochondrial function [35]. Activation of PPARα leads to transcriptional repression of proinflammatory mediators (e.g., TNF-α, COX-2, iNOS) [36] and upregulation of neurotrophic factors such as BDNF [37]. Preclinical evidence suggests that PEA ameliorates neuroinflammatory and behavioral phenotypes in various models of neurodegeneration, including traumatic brain injury, Parkinson’s disease, and AD [32,38,39,40]. In the Tg2576 mouse model, characterized by Aβ overproduction and synaptic loss, PEA reduces gliosis [41] and restores synaptic protein levels [32]. In a previous study, acute PEA administration has been shown to abolish the expression of cocaine-induced CPP [42]. Yet, very little is known about whether PEA influences emotional-motivational behavior or synaptic morphology in the EC and DG, regions critical for affective processing and memory integration. The PPARα-BDNF pathway represents a promising molecular bridge between inflammation resolution and neural plasticity. Activation of PPARα increases BDNF expression in cortical and hippocampal neurons, possibly through direct transcriptional crosstalk with CREB signaling [43,44]. Dysregulation of the PPARα-BDNF signaling axis in depression [45], with restoration by PEA potentially underpinning improved emotional-motivational outcomes. Given that motivational processing and reward-related decision-making involve integration between entorhinal-hippocampal networks and PFC [46,47,48], examining how PEA affects BDNF and PPARα expression in the PFC offers a mechanistic window into the molecular underpinnings of motivational processing in AD.

The present study aims to explore the rewarding and motivational dimensions of AD through a combined behavioral, morphological, and molecular approach in the Tg2576 mice. Specifically, after previous investigation of PEA neuroprotective potential [32], we examined whether chronic administration of PEA via slow-release subcutaneous pellets could (1) affect motivational deficits assessed by the CPP paradigm, (2) modify dendritic spine density in the EC and DG, and (3) alter PPARα and BDNF protein expression in the PFC. We hypothesize that long-lasting, six-month PEA delivery may promote synaptic and neurotrophic recovery through PPARα-mediated BDNF signaling, thereby mitigating motivational impairment linked to PFC-entorhinal pathology in AD.

## 2. Materials and Methods

### 2.1. Animals

All experiments strictly followed the ARRIVE guidelines and complied with the European Directive 2010/63/EU on the protection of animals used for scientific purposes (Protocol Maccarrone 421-2019-PR). The study was conducted in accordance with Italian legislation (L.D. 26/2014) and approved by the Institutional Animal Welfare Committee and the Department of Public Health and Veterinary. Transgenic Tg2576 mice and their wild-type (WT) littermates were bred and maintained in the accredited animal facility of the IRCCS Santa Lucia Foundation (Rome, Italy). Animals were housed in groups of 3–4 per cage at controlled temperature (22–23 °C) and humidity (60 ± 5%), with a 12 h light/dark cycle. Food and water were provided ad libitum throughout the study. Given the increased inter-male aggression characteristic of this strain, which typically begins around 7 months of age [49], Tg2576 mice exhibiting overt aggression were individually housed to ensure animal welfare and minimize social stress.

#### Alzheimer’s Disease-like Mouse Model

Male Tg2576 transgenic mice expressing the human amyloid precursor protein (APP) carrying the Swedish double mutation (K670N/M671L) were used as an AD-like model. This strain displays progressive cognitive deficits, amyloid plaque formation, neuroinflammatory responses, and synaptic deterioration [50]. Tg2576 mice were maintained as heterozygotes by crossing hemizygous males (Tg2576-F0) with C57BL/6J/SJL-F0 hybrid females, obtained by mating SJL males with C57BL/6J WT females. Offspring were genotyped at 20–25 days of age by PCR amplification of genomic DNA extracted from tail biopsies. PCR reactions were performed using 10 ng of DNA per sample and the following primers: forward 5′-CTG ACC ACT CGA CCA GGT TCT GGG T-3′ and reverse 5′-GTG GAT AAC CCC TCC CCC AGC CTA GAC CA-3′ (Sigma Aldrich, St. Louis, MO, USA) as previously reported.

### 2.2. Palmitoylethanolamide (PEA) Chronic Subcutaneous Delivery

Ultra-micronized PEA (UM-PEA) was chronically delivered to Tg2576 mice and their WT counterparts using 90-day sustained-release pellets (Innovative Research of America, Sarasota, FL, USA). The same methodology was adopted in a previous study [32]. Subcutaneous delivery of UM-PEA significantly increased circulating and brain concentrations of PEA in both genotypes. Active and placebo pellets contained identical matrices composed of cholesterol, cellulose, lactose, phosphates, and stearates, differing only by the presence or absence of UM-PEA. According to the manufacturer, the active compound remains dispersed within the matrix in its micrometric crystalline form. Each pellet was formulated to release 80 mg of UM-PEA over 90 days (≈0.88 mg/day, equivalent to 30 mg/kg). The treatment protocol spanned six months, from 6 to 12 months of age, with animals receiving two consecutive subcutaneous implantations (day 0 and day 90) to ensure continuous release for 180 days. Experimental groups were randomly assigned as follows: WT-PEA, WT-Placebo, Tg2576-PEA, and Tg2576-Placebo. To characterize the effects of PEA treatment, mice underwent a multidimensional behavioral test battery covering anxiety-like behavior (Elevated Plus Maze), spatial working memory (Y-maze spontaneous alternation), motor coordination (Rotarod), and Conditioned Place Preference (CPP). Baseline assessments were also conducted during the pre-symptomatic phase at 3 months of age. Detailed Materials and Methods, as well as Results for both the 3-month baseline and the 12-month behavioral testing, are provided in the Supplementary Data (except for CPP, which is presented in the Methods and 12-month results in the main paper). The CPP paradigm used to assess learning of the motivational response (Section 3.4) was followed by histological and biochemical analyses.

### 2.3. Surgical Procedures

Mice were anesthetized via intraperitoneal (i.p.) injection of a solution containing Zoletil 100 (tiletamine HCl 50 mg/mL + zolazepam HCl 50 mg/mL; Virbac, Milan, Italy) and Rompun 20 (xylazine 20 mg/mL; Bayer S.p.A, Leverkusen, Germany). Following induction of anesthesia, the mid-dorsal area (~1 cm^2^) was shaved, disinfected with ethanol, and treated with Betadine. Animals were then positioned in a prone position on a sterile, ethanol-cleaned surgical platform. After a 4–5 mm dorsal incision was made, the pellets were gently placed in the subcutaneous space above the musculature, and then the incision was closed with 9 mm autoclips (Kent Scientific, Torrington, CT, USA). Animals were kept on a heated pad (37 °C) until recovery from anesthesia, housed individually for the first post-surgery 48 h, and then reunited with their cage mates (3–4/group). Precautions were taken to prevent exposure of the pellets to organic solvents or other fluids during implantation, to ensure consistent drug release [51].

### 2.4. Conditioned Place Preference (CPP)

Palatable food-induced CPP was assessed using a custom-built two-chamber apparatus consisting of two Plexiglas compartments (15 × 15 × 20 cm each) distinguished by unique visual and tactile patterns on the floors and walls. The chambers were connected via a central corridor (15 × 5 × 20 cm) equipped with two sliding doors (4 × 20 cm), allowing controlled access. Each compartment contained two black Plexiglas triangular prisms (5 × 5 × 20 cm), arranged in distinct configurations that served as contextual cues. Illumination and environmental features were balanced to minimize any inherent preference for one side of the apparatus. On the pre-conditioning day, each mouse was placed in the central corridor and allowed to freely explore both chambers for 15 min without food. The time spent in each chamber was recorded to assess baseline chamber preference. The conditioning phase consisted of six consecutive daily sessions. During each 30 min session, mice were confined alternately to one of the two chambers: one consistently paired with a palatable food reward (0.5 g milk chocolate; Milka, provided by a food supermarket(Mondelez Italia Services S.r.l., Via Nizzoli 3—20147 Milano, Italy) Alpine Milk Chocolate, 5.31 kcal g^−1^; designated as the paired chamber), and the other chamber paired with regular chow (RC; Mucedola 4RF21 diet; unpaired chamber). Both the chocolate and RC portions were prepared to be isocaloric across sessions. Chocolate consumption was assessed in all groups during the conditioning sessions (see results in the Supplementary Materials). Animals (n = 12 per group) were randomly assigned to receive either chocolate or regular chow, and within each genotype group, the pairing of food type with specific spatial cues was counterbalanced to avoid bias. On the test day (post-conditioning), mice underwent the same free-exploration procedure used during pre-conditioning, and the time spent in each chamber was recorded as a measure of preference. Behavioral activity was captured using a CCD video camera, with signals digitized and analyzed via EthoVision XT 18 software (Noldus Information Technology). The time spent in each compartment during pre- and post-conditioning trials was quantified to calculate CPP preference scores. Chocolate consumption was determined by weighing residual food after each chocolate-paired session and averaging the values across the conditioning phase.

### 2.5. Western Blot Analysis

Frozen brain tissues were homogenized in ice-cold lysis buffer (10 mM EDTA, 50 mM Tris–HCl, pH 7.4, 150 mM NaCl, 1% Triton X-100, Sigma-Aldrich, St. Louis, MO, USA) supplemented with a protease and phosphatase inhibitor cocktail. Samples were subsequently sonicated on ice at approximately 180 W using alternating 10 s sonication and 5 s rest cycles for a total duration of 45 s for each sample. Protein concentrations were determined with the Pierce™ BCA Protein Assay Kits (Thermo Fisher Scientific, Waltham, MA, 02451, USA). Equal amounts of protein (30 μg per sample) were separated on 10% or 12% SDS–polyacrylamide gels and transferred to 0.45 μm or 0.2 μm PVDF membranes (Amersham Biosciences, Piscataway, NJ, USA). Membranes were blocked for 1 h in 5% BSA prepared in Tris-buffered saline containing 0.1% Tween-20 (TBST) and incubated overnight at 4 °C with the following primary antibodies: BDNF (rabbit monoclonal, 1:1000; #ab108319; RRID: AB_10862052; Abcam, Cambridge, UK); PPAR-alpha (rabbit polyclonal, 1:1000; #SAB4502260; RRID: AB_10745197; Sigma Aldrich, St. Louis, MO, USA); GAPDH (mouse monoclonal, 1:5000; #sc-32233; RRID: AB_627679; Santa Cruz Technologies, Dallas, TX, USA). After primary incubation, membranes were washed in TBST and exposed for 1 h at room temperature to the appropriate HRP-conjugated secondary antibodies: goat anti-rabbit (1:5000; #ab6721; RRID: AB_955447; Abcam, Cambridge, UK) and goat anti-mouse (1:5000; #ab6789; RRID: AB_955439; Abcam, Cambridge, UK). Protein bands were visualized using the Lightwave Plus chemiluminescent detection system (GVS, Bologna, Italy), and images were captured with a C-DiGit blot scanner (LI-COR, Lincoln, NE, USA). Band intensities were quantified using Image Studio Software v4.0.21 (LI-COR), and protein expression levels were normalized to GAPDH. When required, membranes were stripped using Mild stripping buffer (0.2 M glycine, 0.1% (*w*/*v*) SDS, 1% (*v*/*v*) Tween-20, adjusted to pH 2.2 with HCl, prepared in deionized water) for 20 min before re-probing. For Western blot analyses, n indicates the number of independent brain lysates (biological samples) available and quantified for the specific target/region, which may vary across proteins due to limited tissue from the relevant brain region. Accordingly, for PPARα expression in PFC: WT placebo, n = 5; WT PEA, n = 6; Tg2576 placebo, n = 4; Tg2576 PEA, n = 5; and for BDNF expression in PFC: WT placebo, n = 6; WT PEA, n = 6; Tg2576 placebo, n = 6; Tg2576 PEA, n = 5.

### 2.6. Morphological Analyses

#### 2.6.1. Golgi-Cox Staining

Golgi-Cox staining of brain tissue was carried out using the FD Rapid GolgiStainTM Kit (FD NeuroTechnologies, Columbia, MD, USA), according to the manufacturer’s instructions. Following sacrifice by cervical dislocation, brains were rapidly removed and immersed in Golgi impregnation solution (A/B) in a plastic container. The impregnation solution was replaced after 24 h, and samples were kept in the dark at room temperature for a total of 14 days. At the end of the impregnation period, brains were transferred to solution C for 72 h, with the solution replaced after 24 h. The brains were then frozen and sliced at 100 μm using a cryostat (CM1860 UV, Leica, Wetzlar, Germany). Sections containing the lateral enthorinal cortex (AP: from −3.0 to −4.0 mm from bregma) and dorsal dentate gyrus (AP: from −1.22 mm to −2.30 mm from bregma) [52] were collected and mounted onto gelatin-coated microscope slides. Mounted sections were rinsed in ddH_2_O for 4 min, then immersed in the D/E staining solution for 10 min, and rinsed again for 4 min. Tissue dehydration was performed through graded ethanol washes (50%, 75%, and 95%) for 4 min each, followed by four immersions in 100% ethanol for 3 min each. Sections were then cleared in xylene (three times, 4 min each) and coverslipped using Eukitt mounting medium. Finally, slides were allowed to dry and stored in the dark at 4 °C.

#### 2.6.2. Analysis of Dendritic Spine Density

**Figure 1:**
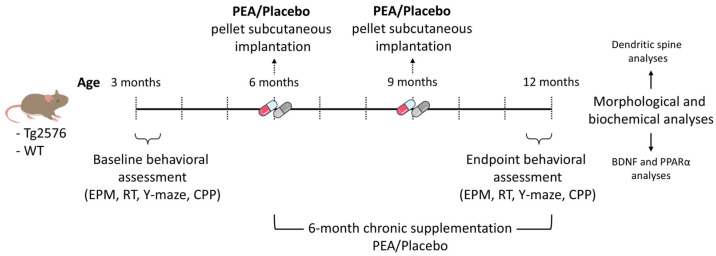
Schematic representation of the experimental timeline. Overview of the study design showing the timing of treatments and the chronological sequence of behavioral testing, followed by tissue collection and downstream morphological and biochemical assessments. All procedures are shown relative to the start of observation/treatment. EPM, Elevated Plus Maze; RT, Rotarod Test; Y-maze, Y-maze Spontaneous Alternation Test; CPP, Conditioned Place Preference.

Quantification of dendritic spine density was performed using an optical microscope (Axio Imager M2, Zeiss, Oberkochen, Germany) equipped with a motorized stage and a camera linked to Neurolucida 2020.1.2 software (MicroBright-Field, 185 Allen Brook Lane, Suite 101 Williston, VT, USA). Dendritic segments with spines were traced using a 100× oil-immersion objective, and the resulting files were subsequently imported into Neurolucida Explorer 2019.2.1 (MicroBright-Field, 185 Allen Brook Lane, Suite 101, Williston, VT, USA) for spine quantitation. Spine density was evaluated in pyramidal neurons of the lateral EC and in granule cells of the dorsal DG. For each experimental group (n = 2 animals/group), twenty dendritic segments (20–25 μm in length) were analyzed per subject in the EC, ten basal and ten apical segments, selected from the distal portions of the dendrites, when applicable (range: 13–20 segments depending on the regional availability of suitable dendritic segments). In the DG, 10 dendritic segments (20–25 µm in length) per subject were collected from the distal dendritic regions. Spine density was calculated as the number of spines divided by the length of each dendrite segment (Figure 1).

### 2.7. Statistical Analysis

Data are presented as box-and-whisker plots in which the central line indicates the median, and the whiskers represent the minimum and maximum values. Statistical analyses were performed using Statistica 12 (StatSoft, Inc., Tulsa, OK, USA, 2014). Graphical representations were generated using GraphPad Prism (version 10.6.1, GraphPad Software, Inc., LLC 225 Franklin Street. Fl. 26. Boston, MA 02110, USA, 2025). In compliance with the European Directive 2010/63/EU for the protection of laboratory animals and the ARRIVE guidelines of the NC3Rs, an a priori power analysis (G*Power v3.1.9.6) was conducted to determine the appropriate sample size based on pilot data. Behavioral data from mice at 3 months of age were analyzed using one-way ANOVA with genotype as the main factor for all parameters, and in the CPP, Elevated Plus Maze, and Rotarod, with a two-way ANOVA with genotype as the between-factor and chamber, arm, or session as the within-factor (repeated measures). Behavioral data from mice at 12 months of age in the CPP, Elevated Plus Maze, and Rotarod tests were analyzed using a three-way mixed-model ANOVA, with genotype and treatment as the between-subject factors and chamber, arm, or session as the within-subject factor (repeated measures). Other experimental data were analyzed by two-way analysis of variance (ANOVA), with genotype and treatment as independent variables. Except for the CPP data at 12 months, behavioral data are described in the Supplementary Materials. Tukey’s post hoc test was applied to assess pairwise differences. Statistical significance was set at *p* < 0.05. The specific statistical tests used for each dataset are indicated in the corresponding figure legends.

## 3. Results

### 3.1. Chronic PEA Delivery Restores CPP in Tg2576 Mice

**Figure 2:**
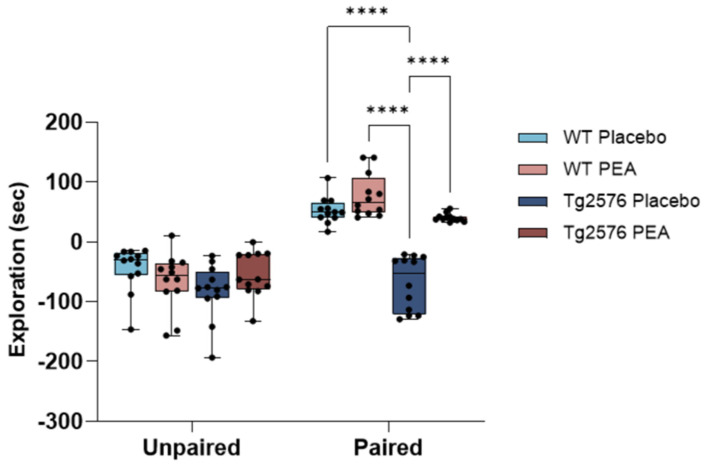
Chocolate-induced CPP from 12-month-old WT and Tg2576 mice following chronic PEA delivery. The figure illustrates the mean difference in time spent in the chocolate-paired and unpaired chambers during the post-conditioning session, calculated as the change relative to the time spent in the corresponding chambers during pre-conditioning (repeated measures ANOVA: treatment, *F*_1,44_ = 22.30, *p* < 0.0001; genotype *F*_1,44_ = 38.16, *p* < 0.0001; treatment × genotype *F*_1,44_ = 20, *p* < 0.0001; chamber, *F*_1,44_ = 124.78, *p* < 0.0001; chamber × treatment, *F*_1,44_ = 16.36, *p* < 0.0001; chamber × genotype *F*_1,44_ = 17.63, *p* < 0.0001; **** *p* < 0.0001 with Tukey’s post hoc test).

Given the central role of reward and motivation processing in AD, we evaluated the behavioral responses of 12-month-old mice using a chocolate-induced CPP response. During the pre-conditioning phase, all animals were allowed to freely explore the two-chamber apparatus. Regardless of genotype, mice spent comparable amounts of time in both compartments, indicating no inherent side bias. Following conditioning with palatable food, WT mice (n =12 per group) exhibited a marked increase in the time spent in the chocolate-paired chamber during the testing phase, confirming the establishment of a reward-associated preference (Figure 2). In contrast, placebo-treated Tg2576 mice (n =12) failed to display place conditioning. Their exploration time remained evenly distributed across chambers during both pre-conditioning and testing sessions, as well as between “unpaired” and “paired” chambers, suggesting an absence of preference toward the chocolate-paired context. This reduction in reward-driven and motivation-driven place preference reflects an impairment in motivational and hedonic processing, as previously observed in this AD-like mouse model [17]. Interestingly, chronic PEA delivery reinstated preference for the chocolate-paired chamber in Tg2576 mice (n = 12) but had no effect in WT mice (n = 12), thereby demonstrating the recovery of motivation-driven place-preference behavior in AD-like mice.

### 3.2. Chronic PEA Delivery Increased PPARα Expression in PFC from Tg2576 Mice

**Figure 3:**
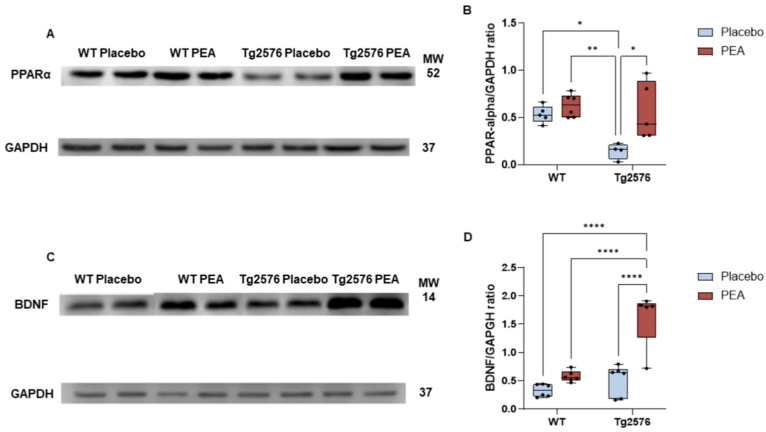
PPARα and BDNF expression in the PFC from 12-month-old WT and Tg2576 mice following chronic PEA delivery. Western blot analyses were performed on PFC extracts from WT and Tg2576 mice; GAPDH served as a loading control. (**A**) Representative immunoblots showing PPARα protein bands in the PFC; MW: molecular weight (kDa). (**B**) Boxplots display the densitometric quantification of PPARα levels normalized to GAPDH. (**C**) Representative immunoblots illustrate BDNF protein bands in the PFC (MW = molecular weight, kDa). (**D**) Boxplots show the densitometric quantification of BDNF expression normalized to GAPDH. The horizontal line within each box indicates the median; individual data points are represented by black circles. Statistical comparisons were conducted using two-way ANOVA followed by Tukey’s post hoc test. Significance levels: * *p* < 0.005; ** *p* < 0.001. **** *p* < 0.0001.

The expression of PPARα protein was evaluated in the PFC of 12-month-old WT and Tg2576 mice following 6-month chronic delivery with PEA or placebo, using Western blot analysis (Figure 3A). Two-way ANOVA showed a significant treatment effect (treatment, F_1,16_ = 10.36, *p* = 0.005; WT placebo: n = 5; WT PEA: n = 6; Tg2576 placebo: n = 4; Tg2575 PEA: n = 5). As illustrated in Figure 3A, PPARα levels were reduced in Tg2576 mice compared with WT (WT placebo vs. Tg2576 placebo * *p* ≤ 0.05 by Tukey’s post hoc). Chronic PEA delivery significantly increased PPARα expression in the PFC from Tg2576 mice (Tg2576 placebo vs. Tg2576 PEA * *p* ≤ 0.05, by Tukey’s post hoc). These findings indicate that chronic PEA delivery elicited the significant expression of PPARα in the PFC from Tg2576 mice.

### 3.3. Chronic PEA Delivery Increased BDNF Expression in PFC from Tg2576 Mice

Using Western blot analysis, BDNF protein expression was quantified in the PFC from 12-month-old WT and Tg2576 mice following chronic six-month infusion with PEA or placebo (Figure 3B). A two-way ANOVA showed a significant main effect of treatment (F_1,19_= 33.03, *p* = 0.0001; a main effect of genotype (F_1,19_ = 27.32, *p* = 0.0001), and a significant treatment × genotype interaction (F_1,19_= 13.22, *p* = 0.005); WT placebo, n = 6; WT PEA, n *=* 6; Tg2576 placebo, n = 6; Tg2576 PEA, n = 5). As depicted in Figure 3B, BDNF expression was markedly increased in Tg2576 PEA mice in comparison to Tg2576 placebo and all WT counterparts (*WT* placebo, *WT PEA*, *Tg2576* placebo vs. *Tg2576 PEA*, **** *p* < 0.0001, by Tukey’s post hoc test). Collectively, these findings suggest that long-term PEA treatment upregulates BDNF expression in the PFC from Tg2576 mice.

### 3.4. Chronic PEA Delivery Restored Dendritic Spine Loss in Tg2576 Mice

**Figure 4:**
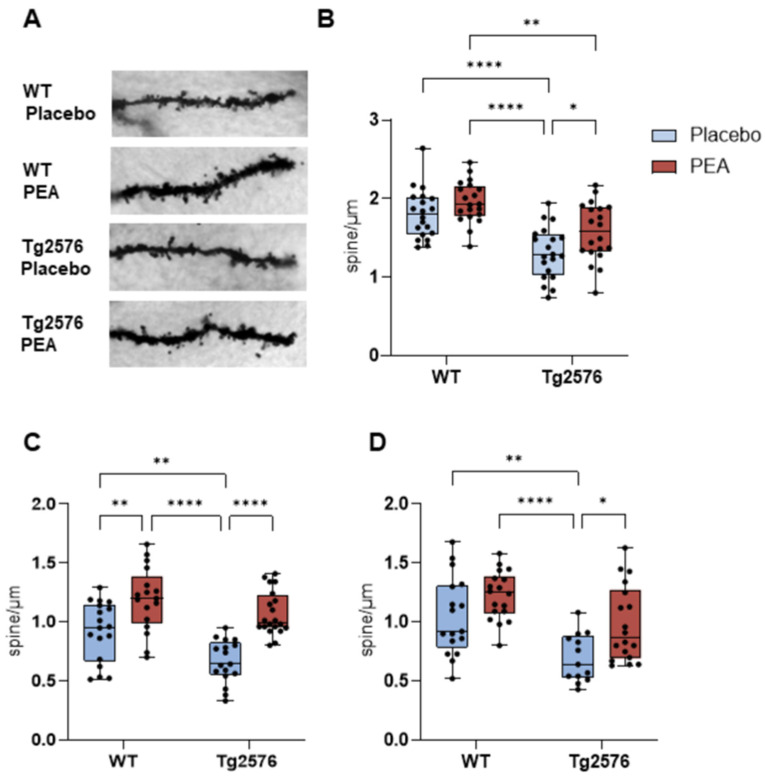
Dendritic spine density in the granule cells of the DG and pyramidal neurons of EC from 12-month-old WT and Tg2576 mice following chronic PEA delivery. (**A**) Representative Golgi-stained images of dendritic spines on DG granule cell dendrites. (**B**) Spine density in the DG (two-way ANOVA followed by Tukey’s post hoc test: * *p* < 0.005; ** *p* < 0.01; **** *p* < 0.0001. (**C**) Spine density in basal dendrites of EC pyramidal neurons (two-way ANOVA followed by Tukey’s post hoc test: ** *p* < 0.01; **** *p* < 0.0001. (**D**) Spine density in apical dendrites of EC pyramidal neurons (two-way ANOVA followed by Tukey’s post hoc test: * *p* < 0.005; ** *p* < 0.01; **** *p* < 0.0001).

To assess whether chronic PEA treatment could restore structural plasticity, we quantified dendritic spine density in granule cells of the DG and pyramidal neurons of EC (Figure 4A).

In the DG, dendritic spine density was analyzed in WT placebo (n = 20 dendritic segments), TG placebo (n = 20 dendritic segments), WT PEA (n = 20 dendritic segments), and TG PEA (n = 20 dendritic segments). Two-way ANOVA revealed significant main effects of genotype (F_1,76_ = 37.41, *p* < 0.0001) and treatment (F_1,76_ = 8.56, *p* < 0.01). Tukey’s multiple comparisons test showed a marked reduction in spine density in Tg2576 placebo mice compared with WT placebo (*p* < 0.0001), and chronic PEA infusion significantly increased spine density in Tg2576 mice (Tg2576 placebo vs. Tg2576 PEA, *p* < 0.05, by Tukey’s post hoc test), restoring values toward WT levels (Figure 4B).

For basal dendrites of the EC, the sample sizes were: WT placebo (n = 18 dendritic segments), TG placebo (n = 17 dendritic segments), WT PEA (n = 18 dendritic segments), and TG PEA (n = 20 dendritic segments). Basal dendritic spine density in the EC displayed significant main effects of genotype (F_1,68_ = 12.48, *p* < 0.001) and treatment (F_1,68_ = 40.23, *p* < 0.0001). Tg2576 placebo mice showed a significant spine deficit in basal dendrites compared with WT placebo (*p* < 0.05). However, chronic PEA treatment robustly increased basal spine density in Tg2576 PEA mice compared with Tg2576 placebo (*p* < 0.0001). Additionally, PEA increased basal spine density in WT mice (*p* < 0.01), indicating a generalized enhancement of basal dendritic structural complexity (Figure 4C).

For apical dendrites of the EC, the sample sizes were: WT placebo (n = 17 dendritic segments), TG placebo (n = 13 dendritic segments), WT PEA (n = 18 dendritic segments), and TG PEA (n = 18 dendritic segments).

Pyramidal apical dendrites of the EC showed a comparable pattern. Two-way ANOVA revealed significant main effects of genotype (F_1,62_ = 18.59, *p* < 0.0001) and treatment (F_1,62_ = 11.41, *p* < 0.01). Tg2576 placebo mice exhibited reduced apical spine density relative to WT controls (*p* < 0.01), and chronic PEA treatment rescued the dendritic spine loss (Tg2576 placebo vs. Tg2576 PEA, *p* < 0.05). In contrast to basal dendrites, apical spine density in WT mice was not significantly affected by PEA (*p* = 0.23) (Figure 4D).

## 4. Discussion

The present study demonstrates that 6-month PEA infusion via sustained-release pellets specifically restores motivational behavior and neuroplasticity in the Tg2576 mouse model of AD. All experimental groups exhibited comparable profiles of anxiety-like behavior, spatial working memory, and motor coordination. By integrating behavioral, molecular, and morphological assessments, our results support the hypothesis that PEA can restore motivational drive by activating the PPARα-BDNF signaling axis, thereby reversing non-cognitive symptoms of AD, such as apathy and deficits in reward processing. Our findings from the CPP task indicate that Tg2576 placebo-treated mice exhibit significant motivational impairments, reflected by reduced exploratory time in the reward-associated compartment and, consequently, by the absence of preference learning.

This interpretation is further supported by the analysis of chocolate consumption during the conditioning phase (see Supplementary Materials). Such an analysis showed that Tg2576 placebo-treated mice, while consuming significantly less chocolate than WT animals, and PEA-treated Tg2576 mice did not completely abstain from eating. The presence of residual consumption indicates conserved engagement with the food stimulus, indicating the preservation of sensory capacity. The reduced intake is consistent with a diminished motivational value assigned to the reward, which would be expected to impair the formation of a stable context–reward association and, consequently, the expression of CPP. These deficits are consistent with the concept of motivational blunting and apathy observed in AD patients [53] and in animal AD models [17,54]. Notably, PEA-treated Tg2576 mice showed robust recovery in CPP behavior, comparable to that of WT mice. This suggests that PEA restores reward-related behavior, likely by restoring prefrontal-EC-hippocampal circuit dynamics. Given the role of the EC, PFC, and hippocampus in integrating motivational drive, behavioral flexibility, goal-directed behavior, and the impact of emotional processing on learning and memory [55,56], these results highlight the capacity of PEA to reinstate functional integrity within this motivational network.

At the molecular level, we observed that Tg2576 mice displayed decreased PPAR-α expression, confirming prior evidence of lipid signaling dysregulation in AD models [32,38,57,58], and in particular the role of PPAR-α in regulating the non-amyloidogenic pathway (i.e., α-secretase) of APP cleavage, and downregulating substrates for β-secretase (BACE1) [32,59]. Long-lasting PEA infusion restored PPAR-α levels in Tg2576 mice, consistent with its role as a direct PPAR-α agonist. Interestingly, while BDNF levels were not significantly reduced in Tg2576 placebo mice, PEA significantly elevated BDNF expression in PEA-treated Tg2576. This suggests that PEA can induce a selective BDNF upregulation and produce a neurotrophic potentiation, which may bolster synaptic resilience and neuroplasticity. These results align with prior evidence that PPAR-α activation promotes BDNF transcription not only via CREB-dependent pathways [60], but also via the activation of PPAR-α-dependent pathways, which may contribute to experience-dependent synaptic remodeling and circuit reorganization in cortico-limbic networks.

Morphological analyses further revealed that Tg2576 mice exhibited reduced dendritic spine density in the DG and in pyramidal neurons of EC. In the EC, spine loss affected both basal and apical dendrites, structures critical for excitatory input integration and synaptic plasticity. Notably, PEA infusion in Tg2576 mice led to an increase in DG spine density; however, this effect was insufficient to restore spine density to levels observed in WT mice. Similarly, chronic PEA infusion increased dendritic spine density on apical dendrites of the EC in Tg2576 mice, resulting in spine densities comparable to those observed in both placebo- and PEA-treated WT mice. Interestingly, PEA infusion promoted an increase in dendritic spines in the EC of both WT and Tg2576 mice, suggesting a broader effect of PEA on basal dendritic plasticity regardless of genotype, and facilitation of dendritic complexity even in the absence of overt pathology. These dendritic compartment-specific effects of PEA may reflect the distinct physiological roles and synaptic input patterns of apical and basal dendrites. Apical dendrites, which extend into Layer I and receive feedback and modulatory inputs from higher-order regions such as the hippocampus and PFC, are particularly involved in sensory gating, feedback integration, and memory consolidation. Thus, the partial rescue of apical spine density in Tg2576 mice may support a limited recovery of top-down integrative functions, including the plasticity of excitatory synapses involved in executive control and motivational salience. In contrast, basal dendrites, which receive local feedforward inputs within deeper cortical layers, are implicated in fine-grained, spatially resolved processing and may contribute to stable representations of contextual information. Together, this dendritic architecture supports non-linear integration, dendritic spikes, and compartmentalized plasticity, which are essential for synaptic plasticity and thus for the encoding of behaviorally relevant experiences.

The consistent increase in dendritic spine density across genotypes suggests that PEA may exert more robust effects on local circuit integration and spatial coding, potentially contributing to the stabilization of EC-driven computations disrupted in Tg2576 mice. In line with this interpretation, our behavioral data showed that in Tg2576 mice, chronic PEA infusion restored CPP responses to reward. The reinstatement of CPP may reflect improved EC-mediated context-reward association. The EC has been shown to contribute to the integration of spatial and contextual representations with salient outcomes, and entorhinal neurons exhibit reward-modulated activity during navigation tasks [61]. Indeed, multiple studies indicate that the EC is not merely a spatial mapping hub, but also plays a direct role in encoding context–reward associations. For example, Butler and colleagues demonstrated that EC spatial maps reorganize around learned reward sites [62]. Moreover, input from EC layer 3 drives hippocampal place cell plasticity during reward location learning [63], and EC neurons can represent not only spatial position but also task-relevant remote reward locations [64]. These findings support the idea that the observed increase in basal dendritic spine density in the EC may underlie the restoration of CPP by enhancing local feedforward microcircuits that encode the rewarding context. In this view, EC contributes to linking contextual representations with reward retrieval, as occurs during CPP performance. Therefore, remodeling of spine density of EC basal dendrites, induced by PEA treatment, may facilitate the encoding and retrieval of context-reward associations (such as chocolate CPP) by strengthening local feedforward processing and its projections to the hippocampus and reward-related circuits. Moreover, the trophic effect of PEA on dendritic complexity may reflect BDNF-mediated enhancement of spine density, which is essential for experience-dependent plasticity and reward-related learning. There is, for example, evidence that BDNF/TrkB signaling in the nucleus accumbens mediates cocaine-induced spine increases and associated CPP [65]. Moreover, neurotrophin-driven synaptic structural changes in the PFC underpin food and drug-reinforced behavior and reward-related decision making [66], supporting the view that BDNF fosters structural plasticity in motivational contexts. Thus, our observation that PEA enhances dendritic complexity may reflect a restoration of the structural substrate, for context–reward integration within EC microcircuits, via BDNF signaling.

Taken together, these findings support a model in which PEA acts via PPAR-α activation to enhance BDNF signaling, leading to morphological plasticity in EC and PFC circuits. This remodeling likely underpins the recovery of motivational behavior, as the EC-prefrontal loop is essential for encoding the salience and value of reward-predictive cues [61,67]. The fact that PEA restored both behavioral and dendritic phenotypes further strengthens the view that synaptic morphology and motivation can be functionally coupled in early AD. These results shift the focus from purely cognitive deficits to a broader emotion-motivation circuitopathy and highlight PEA as a lipid-based neuromodulator that may restore both function and form.

This integrative framework highlights PEA as a lipid-based neuromodulator capable of restoring morphological and functional plasticity in circuits underlying motivational processing. By shifting the focus from cognitive to reward-motivation networks, this study seeks to broaden our understanding of AD as a disorder of affective disconnection, where neuroinflammation, synaptic loss, and motivational deficits converge. Within this framework, the study supports the view that therapeutically targeting neurotrophic and lipid signaling pathways, such as the PEA-PPARα-BDNF axis, may offer novel routes for preserving both synaptic integrity and motivational response in AD.

### Limitations

While these findings are promising, several limitations should be considered. First, the study did not assess potential sex differences in response to PEA, despite well-documented sex-specific trajectories in AD progression. We planned to conduct a future study to address this interesting issue. Second, although our analysis focused on the PFC, EC, and DG regions, which are critically involved in reward processing and motivation, future studies should extend this work to include other key structures, such as the amygdala and ventral striatum. Third, the morphological analysis of dendritic spine density was performed on a relatively small number of animals, which may limit the generalizability of the findings. Therefore, confirmation in a larger cohort would be desirable. Finally, the long-term impact of PEA remains to be determined; longitudinal studies are needed to evaluate whether its effects persist beyond the treatment window and whether they influence the trajectory of neurodegeneration.

## 5. Conclusions

Our findings support the PEA-PPARα-BDNF pathway as a promising therapeutic target for addressing motivational and affective symptoms of AD. By enhancing BDNF signaling in the prefrontal cortex, PEA restores key aspects of activity-dependent synaptic plasticity disrupted in early AD. The concurrent recovery of motivational behavior and structural integrity suggests a functional link between synaptic morphology and reward-related processing. These results highlight the potential of PEA as a lipid-based neuromodulator capable of reinstating adaptive plasticity within emotion-motivation networks. Future studies should assess long-term efficacy, sex differences, and broader circuit-level effects. Still, this work offers a proof-of-concept for targeting synaptic plasticity mechanisms to restore non-cognitive function in AD.

## Data Availability

The raw data supporting the conclusions of this article will be made available by the authors on request.

## Supplementary Materials

The following supporting information can be downloaded at: https://www.mdpi.com/article/10.3390/cells15080669/s1, Figure S1: Elevated Plus Maze of WT and Tg2576 mice at 3 (A,B) and 12 (C,D) months of age. In this and in the following figures: box plots—horizontal line and + for median and mean; bottom and top edges for 25th and 75th percentiles; whiskers for extreme data points. Duration of time spent in arms (A,C) and frequency of total entries in arms (B,D) of WT and Tg2576 (#: arm effect, *p* < 0.000001; *: interaction effect, *p* < 0.05). Figure S2: Rotarod Test of WT and Tg2576 mice at 3 and 12 months of age. Mean latency to fall off the apparatus during the training phase (A,C) and highest latency to fall off the apparatus in the test phase (B,D) (#: session effect, *p* < 0.000001). Figure S3: Y-maze Spontaneous Alternation Test of WT and Tg2576 mice at 3 and 12 months of age. Spontaneous Alternations (A,C) and Frequency of total entries in arms (B,D). Figure S4: Chocolate-induced CPP from 3-month-old WT and Tg2576 mice. Figure S5: Chocolate consumption during CPP conditioning sessions. References [68,69,70,71] are cited in Supplementary Materials.

## Abbreviations

The following abbreviations are used in this manuscript:

AD	Alzheimer’s Disease
Aβ	Amyloid-beta
APP	Amyloid Precursor Protein
ARRIVE	Animal Research: Reporting of In Vivo Experiments
BACE1	Beta-site APP-Cleaving Enzyme 1
BDNF	Brain-Derived Neurotrophic Factor
COX-2	Cyclooxygenase-2
CPP	Conditioned Place Preference
CREB	cAMP Response Element-Binding Protein
DG	Dentate Gyrus
EC	Entorhinal Cortex
EDTA	Ethylenediaminetetraacetic Acid
EU	European Union
GAPDH	Glyceraldehyde-3-Phosphate Dehydrogenase
HRP	Horseradish Peroxidase
i.p.	Intraperitoneal
iNOS	Inducible Nitric Oxide Synthase
LD	Legislative Decree
MW	Molecular Weight
NAE	*N*-acylethanolamine
PEA	Palmitoylethanolamide
PFC	Prefrontal Cortex
PPARα	Peroxisome Proliferator-Activated Receptor Alpha
PVDF	Polyvinylidene Difluoride
RC	Regular Chow
SDS	Sodium Dodecyl Sulfate
TrkB	Tropomyosin receptor kinase B
WT	Wild-Type
