# Supplementary material for: Sustained Palmitoylethanolamide Infusion Restores Incentive Motivation and Synaptic Plasticity in the Tg2576 Mouse Model of Alzheimer’s Disease
Source: Cells. 2026 Apr 9;15(8):669. doi: 10.3390/cells15080669 (PMC13114631; doi:10.3390/cells15080669)
Supplement: Supplementary file 1 [file cells-15-00669-s001.zip › cells-4135317-supplementary.pdf]

Supplementary Materials

Behavioral Analyses of Tg2576 and WT Mice at 3 and 12 Months of Age

Elevated Plus Maze (EPM) Test

Materials and Methods

EPM is a validated test to measure anxiety and locomotor activity in rodents based on their natural proclivity toward dark and enclosed spaces and due to their natural aversion to open spaces [57,58]. The maze consisted of a wooden cross-shaped structure elevated 60 cm above the floor, with a central platform (5 x 5 cm) and four arms of 30 cm length and 5 cm width each. Two opposing arms were enclosed by walls 15 cm high, while the other two oppositely positioned open arms had no walls. During a 5 min trial, the mouse was placed in the central platform and allowed to freely explore the maze. Animal performance was video-recorded by a ceiling-mounted camera and manually scored by an operator blind to mice experimental grouping by using EthoVision XT (Noldus, Wageningen, The Netherlands). The maze was cleaned with a 10% ethanol solution between trials to remove olfactory cues.

The EPM parameters considered were: duration of time spent, and total frequency of entries in the open and closed arms.

Results

At 3 months of age, all mice spent significantly more time in the closed arms compared to the open arms ($p < 0.000001$), with no significant differences between WT and Tg2576 groups in the duration of time spent in open or closed arms (Fig S1A). Regarding locomotor activity, no differences between groups were noted in the total entries across all arms (Fig. S1B). Moreover, all mice entered significantly more times the closed arms compared to the open arms ($p < 0.000001$) (Fig. S1B). These results indicate similar levels of anxiety and locomotor activity across all animals.

At 12 months of age, a three-way ANOVA for repeated measures was used to compare differences among treatment (placebo or PEA), genotype (WT or Tg2576), and arm (open or closed). No significant differences were observed in the duration of time spent in closed vs. open arms, indicating similar anxiety-like behavior among all groups. In fact, all mice spent significantly more time in the closed arms compared to the open arms ($p < 0.000001$) (Fig 1A). Furthermore, all mice entered significantly more times in the closed arms compared to the open arms ($p < 0.000001$) (Fig. S1C, D). Significant arm x genotype ($p = 0.0215$) and arm x genotype x treatment ($p = 0.0277$) interactions were found in total entries, showing a significant increase in locomotor activity in all Tg2576 mice.

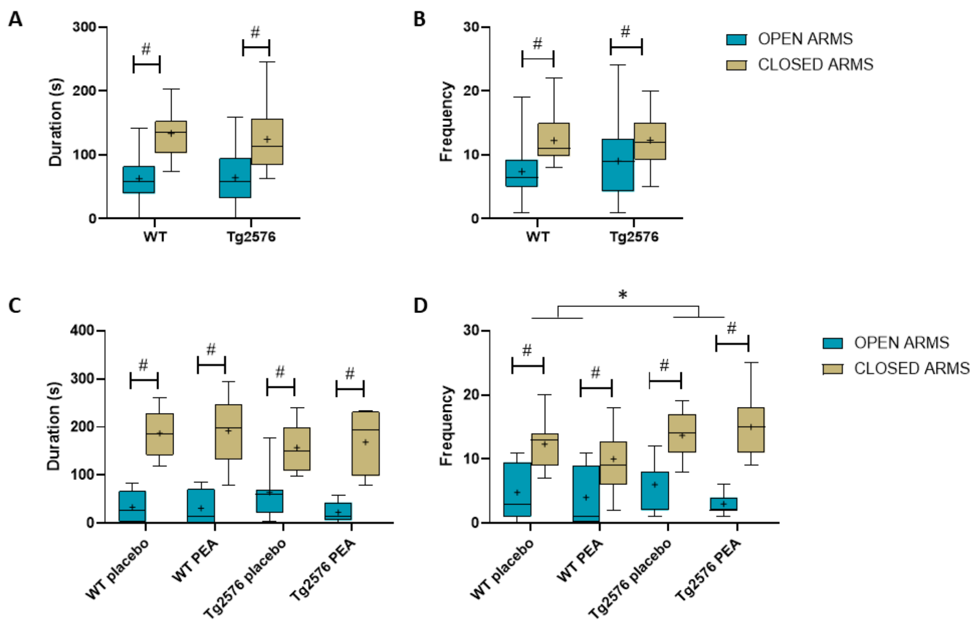

Figure S1. Elevated Plus Maze of WT and Tg2576 mice at 3 (A, B) and 12 (C, D) months of age. In this and in the following figures: box plots—horizontal line and + for median and mean; bottom and top edges for 25th and 75th percentiles; whiskers for extreme data points. Duration of time spent in arms (A, C) and frequency of total entries in arms (B, D) of WT and Tg2576 (#: arm effect, $p < 0.000001$; *: interaction effect, $p<0.05$).

Rotarod Test (RT)

Materials and Methods

RT is a validated test used to assess motor coordination and balance in rodents. The apparatus consisted of a computerized, electronically controlled system comprising a four-line suspended rotating drum (3 cm diameter), separated by plastic panels to prevent mice from running into the adjacent line and from disturbing each other.

The apparatus was set up in an environment with minimal disturbances. Each animal was placed on the rotating lane, and when it dropped onto the platform below its lane, the latency to fall (seconds) was automatically recorded. The original protocol [59,60] was modified, and it consisted of a linear acceleration rate (4-40 rpm) for a maximum of 300 seconds.

Mice were acclimated to the rod at 4 rpm for 30 sec on the first day of the test. Then, 4 sessions comprising three trials, separated by 5 min inter-trial (ITI), were performed on two consecutive days, with at least 3 h between sessions (so, a morning session followed by an afternoon session for two days). The first 3 sessions were considered the training phase. The session performed in the afternoon of the second day was considered the test phase. The apparatus was cleaned with a 10% ethanol solution between trials to remove olfactory cues.

The RT parameters considered were mean latency to fall off the apparatus during the training (to evaluate the learning curve) and the highest latency to fall during the test session (to assess motor coordination).

Results

At 3 and 12 months of age, a significant effect of the session ($p < 0.000001$) on mean latency to fall off during the training phase (Fig. S2A, C) and no phase differences among WT and Tg2576 groups in the highest latency to fall off the apparatus (Fig. S2B, D) were found, indicating a similar learning curve and intact motor coordination in all mice.

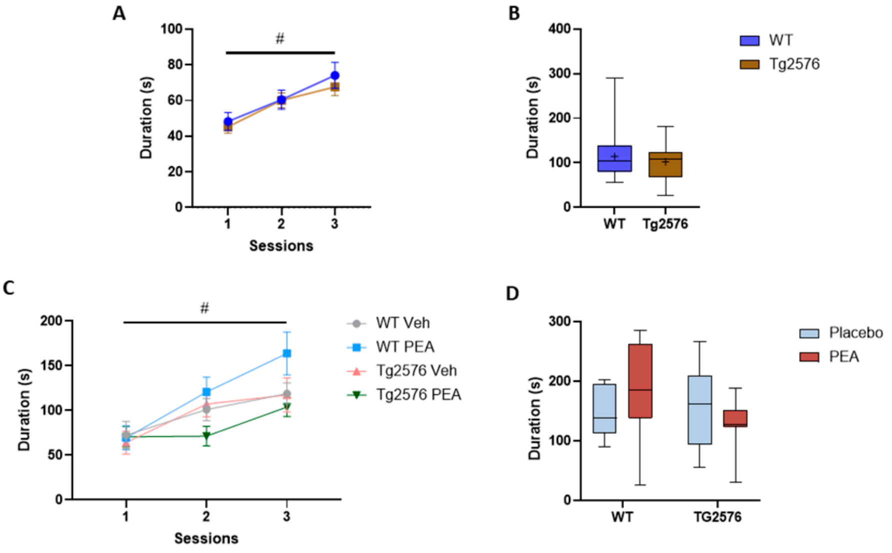

Figure S2. Rotarod Test of WT and Tg2576 mice at 3 and 12 months of age. Mean latency to fall off the apparatus during the training phase (A, C) and highest latency to fall off the apparatus in the test phase (B, D) (#: session effect, $p < 0.000001$).

Y-maze Spontaneous Alternation Test

Materials and Methods

The Y-maze is a behavioral test to evaluate spatial working memory. It is based on the willingness of rodents to explore new environments. In fact, rodents tend to investigate a new arm of the maze rather than return to one that was previously visited.

Y maze consists of an opaque plastic Y-shaped apparatus with 120° angles. When animals were 3-month-old, the apparatus was white, and each arm of the apparatus was 37 cm long, 12.5 cm wide, and 15 cm high; when the animals were 12-month-old, the maze was grey, and each arm of the apparatus was 37.5 cm long, 12 cm wide, and 14 cm high. A mouse was considered inside an arm only if all four limbs were within it. Animals' performances were video-recorded by a ceiling-mounted camera and scored. The test trial lasted 10 minutes. The apparatus was cleaned with a 10% ethanol solution between trials to remove olfactory cues.

Each arm was named with a letter (A, B, or C) in order to assess the percentage of alternations, calculated as the number of alternations (entries into three different arms, consecutively) divided by the total possible alternations (i.e., the total number of arms entered minus 2) and multiplied by 100 [61]. For example, if the order of arm entries was: ABCCBABCABC, the researcher would score a total of 6 spontaneous alternations (in the order: ABC, CBA, ABC, BCA, CAB, ABC). With a total of 11 arm entries, the spontaneous alternation percentage would be 67% [62].

The parameters considered were: percentage of spontaneous alternations and total number of entries in arms.

Results

At 3 and 12 months of age, no significant differences among groups in spontaneous alternations (Fig. S3A, C) and total entries into arms (Fig. S3B, D) were observed, indicating similar spatial working memory performances.

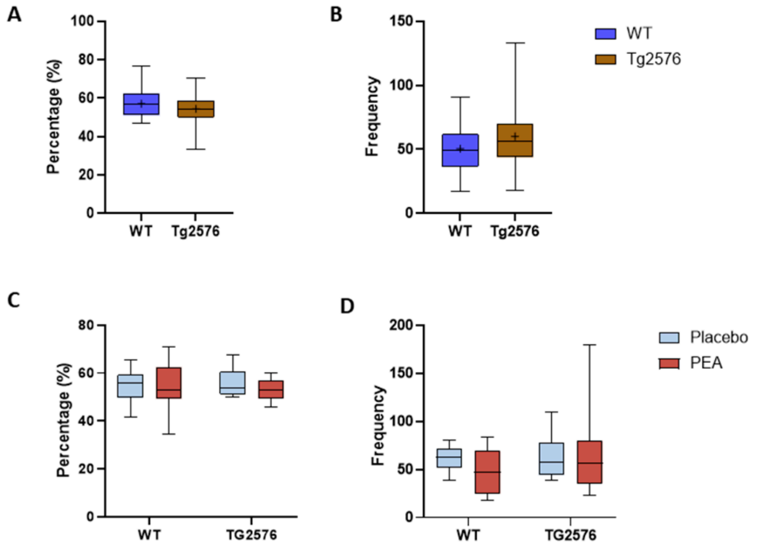

Figure S3. Y-maze Spontaneous Alternation Test of WT and Tg2576 mice at 3 and 12 months of age. Spontaneous Alternations (A, C) and Frequency of total entries in arms (B, D).

Conditioned Place Preference

Materials and Methods

As described in the main paper.

Results

After conditioning with a palatable food, both WT and Tg2576 mice showed a significant increase in the time spent in the chamber associated with chocolate during the testing phase, indicating successful formation of a reward-based preference (Figure S4). This suggests that at 3 months of age, both genotypes were equally capable of forming place preference toward the chocolate-associated context.

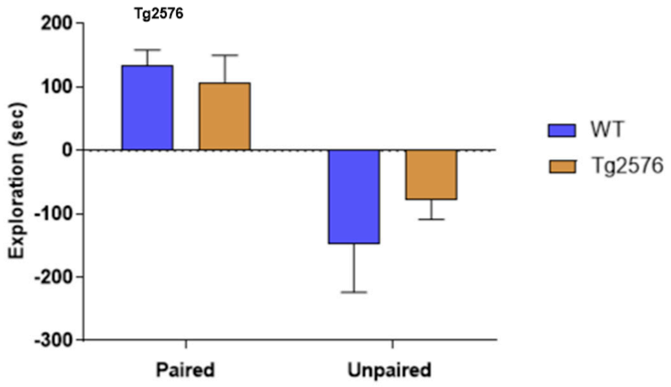

Figure S4. Chocolate-induced CPP from 3-month-old WT and Tg2576 mice.

The figure displays the average difference in time spent between the chocolate-paired and unpaired chambers during the post-conditioning session, computed as the change relative to the time spent in each respective chamber during the pre-conditioning phase.

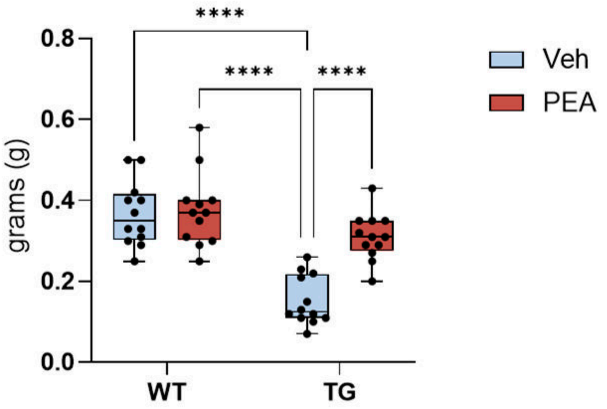

Figure S5. Chocolate consumption during CPP conditioning sessions. (N = 12 per group: WT Placebo, WT PEA, Tg2576 Placebo, Tg2576 PEA). Data were analyzed by two-way ANOVA followed by Tukey's post hoc test (****$p < 0.0001$).

Raw, uncropped, original Western blot images displayed in Figure 3A/C

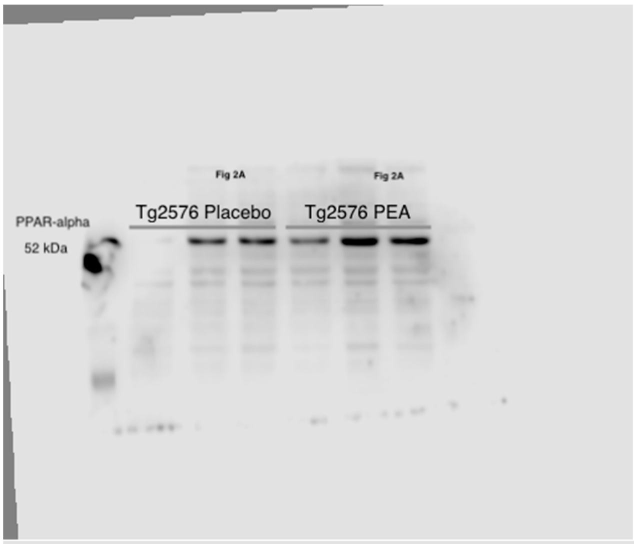

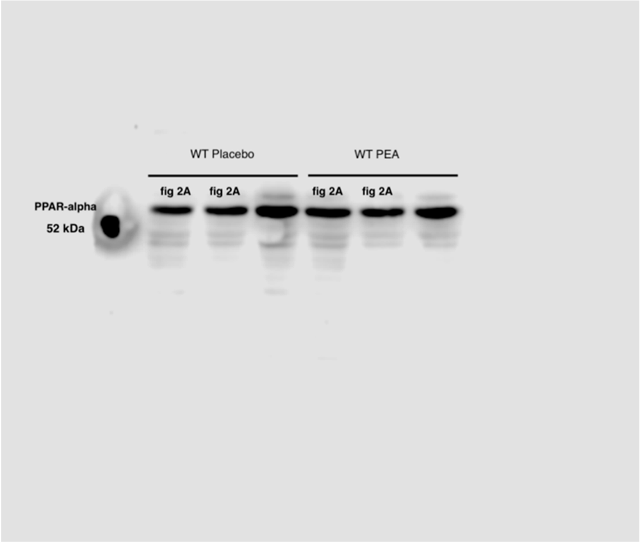

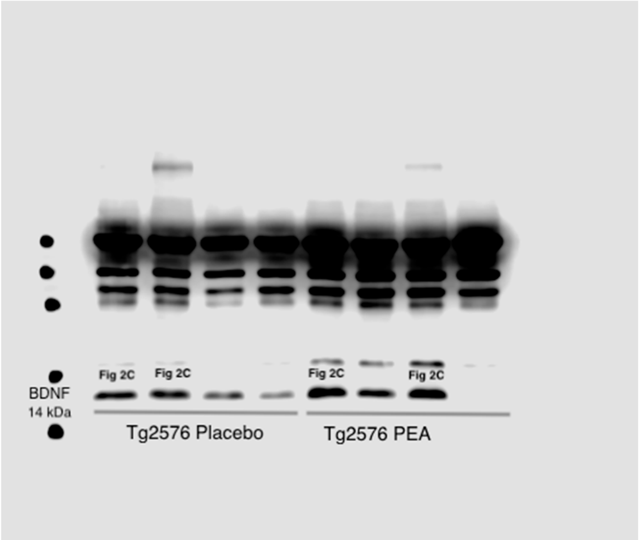

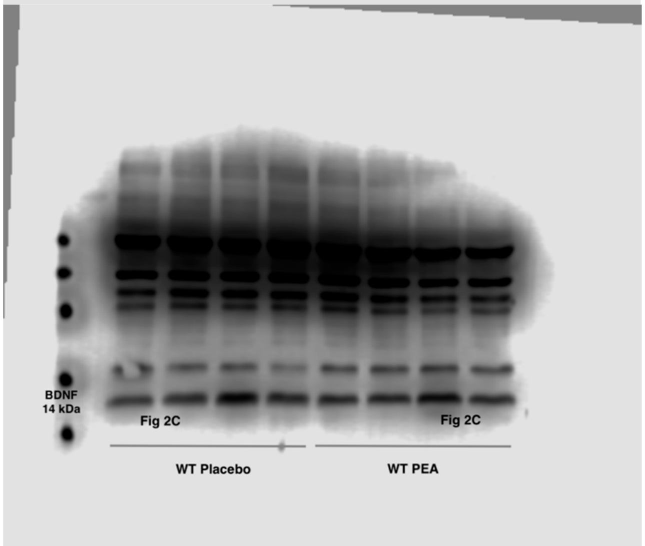

References

57. Cutuli, D.; Pagani, M.; Caporali, P.; Galbusera, A.; Laricchiuta, D.; Foti, F.; Neri, C.; Spalletta, G.; Caltagirone, C.; Petrosini, L.; et al. Effects of Omega-3 Fatty Acid Supplementation on Cognitive Functions and Neural Substrates: A Voxel-Based Morphometry Study in Aged Mice. *Front. Aging Neurosci.* **2016**, *8*, 171975, doi:10.3389/FNAGI.2016.00038/BIBTEX.
58. Ruehle, S.; Remmers, F.; Romo-Parra, H.; Massa, F.; Wickert, M.; Wörtge, S.; Häring, M.; Kaiser, N.; Marsicano, G.; Pape, H.C.; et al. Cannabinoid CB1 Receptor in Dorsal Telencephalic Glutamatergic Neurons: Distinctive Sufficiency for Hippocampus-Dependent and Amygdala-Dependent Synaptic and Behavioral Functions. *J. Neurosci.* **2013**, *33*, 10264–10277, doi:10.1523/JNEUROSCI.4171-12.2013.
59. Sciamanna, G.; Ponterio, G.; Vanni, V.; Laricchiuta, D.; Martella, G.; Bonsi, P.; Meringolo, M.; Tassone, A.; Mercuri, N.B.; Pisani, A. Optogenetic Activation of Striatopallidal Neurons Reveals Altered HCN Gating in DYT1 Dystonia. *Cell Rep.* **2020**, *31*, doi:10.1016/J.CELREP.2020.107644.
60. Massaro Cenere, M.; Tiberi, M.; Paldino, E.; D'Addario, S.L.; Federici, M.; Giacomet, C.; Cutuli, D.; Matteocci, A.; Cossa, F.; Zarrilli, B.; et al. Systemic Inflammation Accelerates Neurodegeneration in a Rat Model of Parkinson's Disease Overexpressing Human Alpha Synuclein. *npj Park. Dis. 2024 101* **2024**, *10*, 213-, doi:10.1038/s41531-024-00824-w.
61. Sarnyai, Z.; Sibille, E.L.; Pavlides, C.; Fenster, R.J.; McEwen, B.S.; Tóth, M. Impaired Hippocampal-Dependent Learning and Functional Abnormalities in the Hippocampus in Mice Lacking Serotonin1A Receptors. *Proc. Natl. Acad. Sci. U. S. A.* **2000**, *97*, 14731–14736, doi:10.1073/PNAS.97.26.14731/ASSET/7F4C1003-D7A4-4754-A46E-DA9B219A5459/ASSETS/GRAPHIC/PQ2605055005.JPEG.
62. Miedel, C.J.; Patton, J.M.; Miedel, A.N.; Miedel, E.S.; Levenson, J.M. Assessment of Spontaneous Alternation, Novel Object Recognition and Limb Clasping in Transgenic Mouse Models of Amyloid-β and Tau Neuropathology. *J. Vis. Exp.* **2017**, *2017*, doi:10.3791/55523.
